# Supplementary material for: Nanocarrier mediated delivery of insecticides into tarsi enhances stink bug mortality
Source: Nat Commun. 2024 Nov 11;15:9737. doi: 10.1038/s41467-024-54013-7 (PMC11554816; doi:10.1038/s41467-024-54013-7)
Supplement: Supplementary file 1 — Supplementary Information [file 41467_2024_54013_MOESM1_ESM.pdf]

Nanocarrier mediated delivery of insecticides into tarsi enhances stink bug mortality

Sandeep Sharma[1], Thomas M. Perring[2], Su-Ji Jeon[1], Huazhang Huang[3], Wen Xu[3], Emir Islamovic[3], Bhaskar Sharma[1], Ysabel Milton Giraldo[2], and Juan Pablo Giraldo[1*]

1. Department of Botany and Plant Sciences, University of California, Riverside, California 92521, United States
2. Department of Entomology, University of California, Riverside, California 92521, United States
3. BASF corporation, 26 Davis Drive, North Carolina 27709-3528, United States

*Corresponding author

email: juanpablo.giraldo@ucr.edu

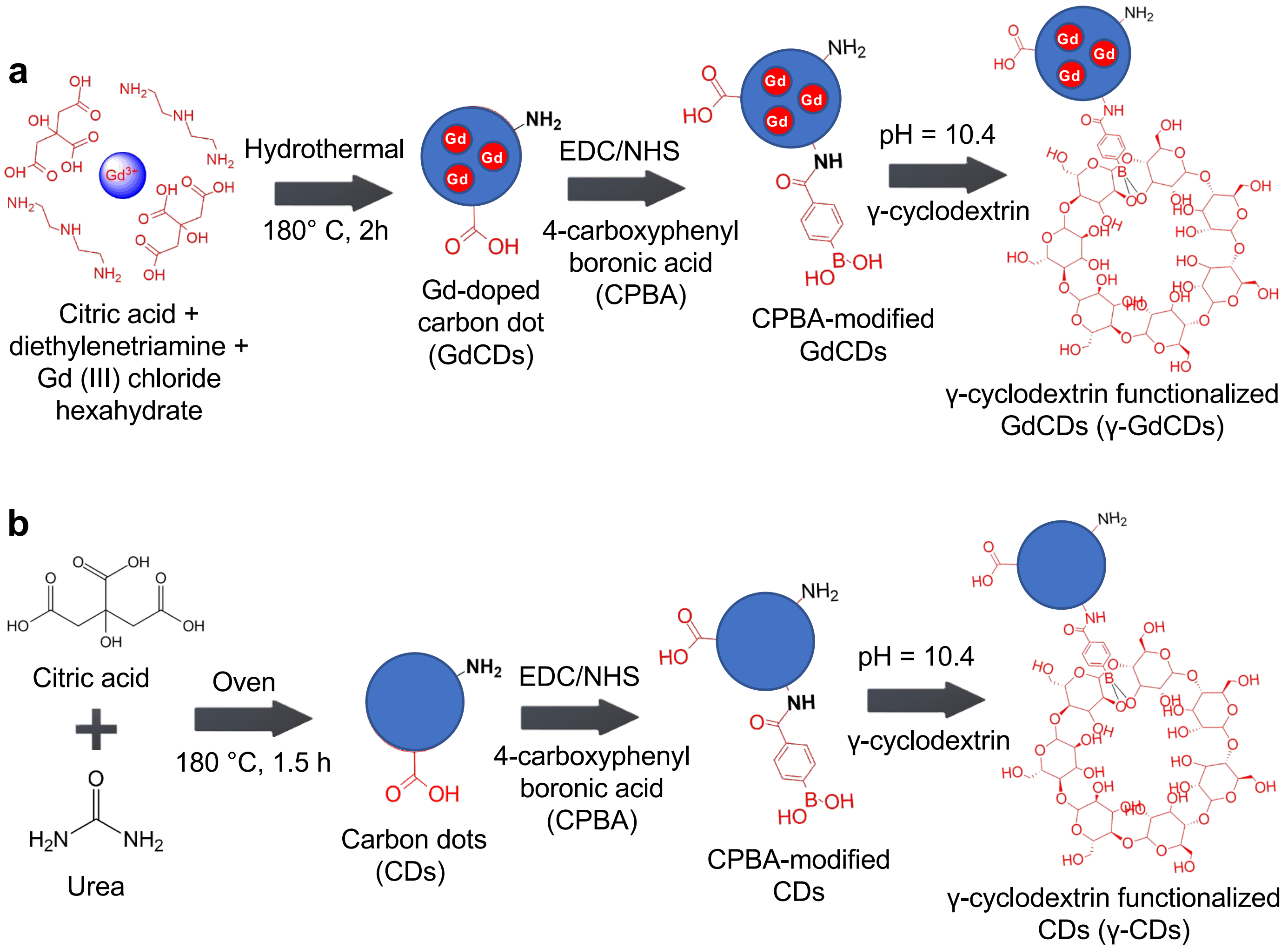

Supplementary Figure 1. **Synthesis of nanocarriers.** Schematic depicts the stepwise synthesis of **a)** γ-cyclodextrin modified GdCDs (γ-GdCDs), and **b)** γ-cyclodextrin modified CDs (γ-CDs).

Material	Market price	Company	Website	γ-CDs nanocarrier synthesis per hectare
Urea	$3.9 per Kg	Duda Energy LLC	https://dudadiesel.com/choose_item.php?id=urea45	$40.6
Citric acid	$6.6 per Kg	Duda Energy LLC	https://dudadiesel.com/choose_item.php?id=50ca	$40.16
3-(3-Dimethylaminopropyl)-1-ethyl-carbodiimide hydrochloride (EDC)	$352.8 per Kg	Chemimpex.com	https://www.chemimpex.com/3-3-dimethylaminopropyl-1-ethyl-carbodiimide-hydrochloride	$24.7
N-Hydroxysuccinimide (NHS)	$39 per Kg	Chemimpex.com	https://www.chemimpex.com/n-hydroxysuccinimide	$1.95
γ-cyclodextrin	$397.4 per Kg	Chemimpex.com	https://www.chemimpex.com/category/search/cyclodextrins/2691	$20.4
**Subtotal** (Precursors and reagents)				**$128**
Dialysis membrane	$13.4 per m	AliExpress.com	https://www.aliexpress.us/item/3256802665108380.html?	$450
**Total** (including purification material)				**$578**

Supplementary Table 1. Cost analysis for γ-CDs nanocarrier synthesis. Estimated cost of precursors and chemical reagents for nanocarrier synthesis is approximately ~$130 per hectare in a field having 325,000 crop plants ($0.0004 per plant) with a recommended 12-inch row spacing. Adding purification costs using a dialysis membrane, increases the nanocarrier cost to ~$580 per hectare. However, dialysis membranes can be reused and diafiltration large-scale purification techniques (widely used in industry) can replace dialysis. Significant cost reductions of perhaps an order of magnitude could be achieved by purchasing materials in bulk and performing large-scale synthesis.

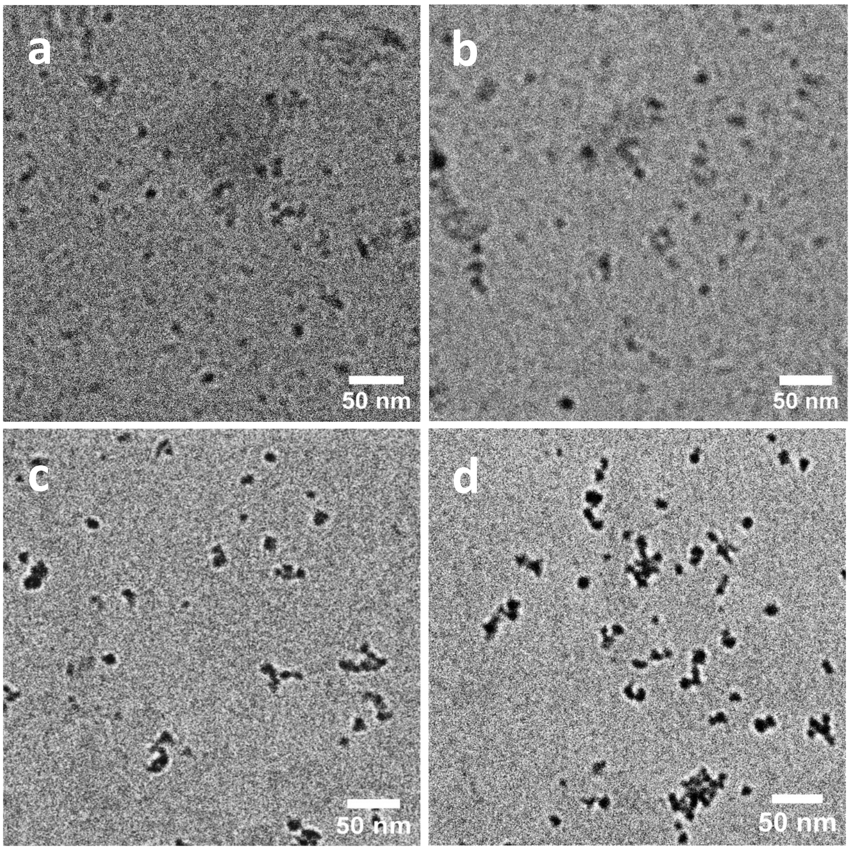

Supplementary Figure 2. **Morphological characterization of nanocarriers**. TEM images of **a**) CDs, **b**) γ-CDs, **c**) GdCDs, and **d**) γ-GdCDs. The size of CDs and GdCDs after modification with γ-cyclodextrin was significantly increased from 5.6 ± 1 to 9.0 ± 1.3 nm ($P < 0.0001$) and 6.1 ± 1.3 to 9.3 ± 1.5 nm ($P < 0.0001$), respectively ($n = 50$).

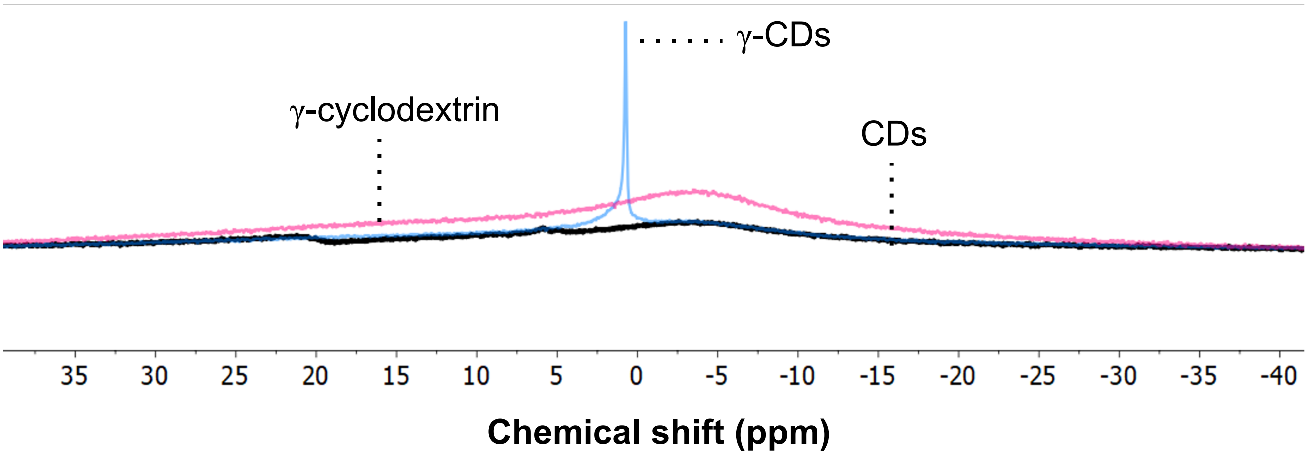

Supplementary Figure 3. ^{11}B NMR spectra of CDs, γ-cyclodextrin, and γ-CDs. γ-CDs spectra showed an intense boron peak arising from the boronic ester bond formed between CDs and γ-cyclodextrin; whereas no boron peak was observed in the spectra of CDs and γ-cyclodextrin.

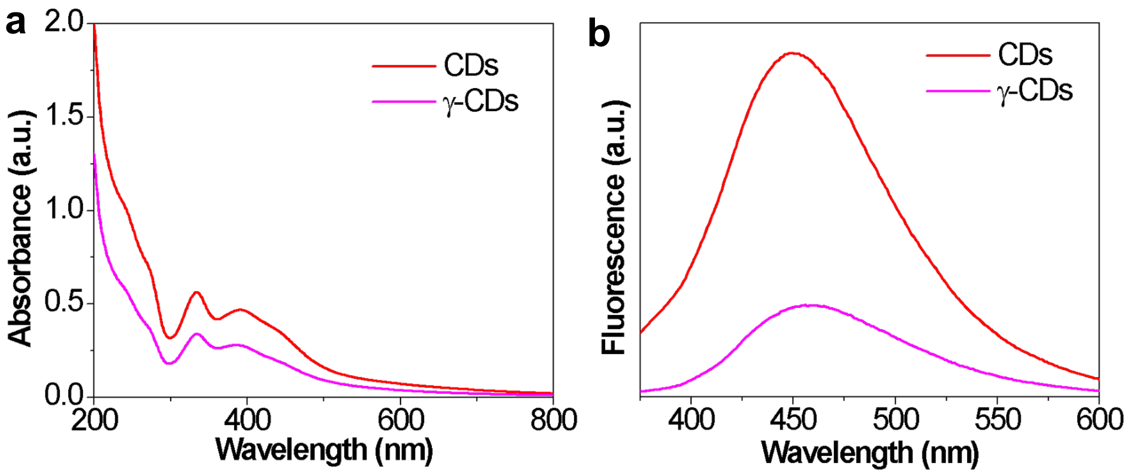

Supplementary Figure 4. Optical properties of CDs and γ-CDs. a) Absorbance spectra of CDs and γ-CDs showed two absorbance peaks at 334 nm and 394 nm. **b)** Fluorescence spectra of CDs and γ-CDs showed fluorescence emission maxima at 450 nm.

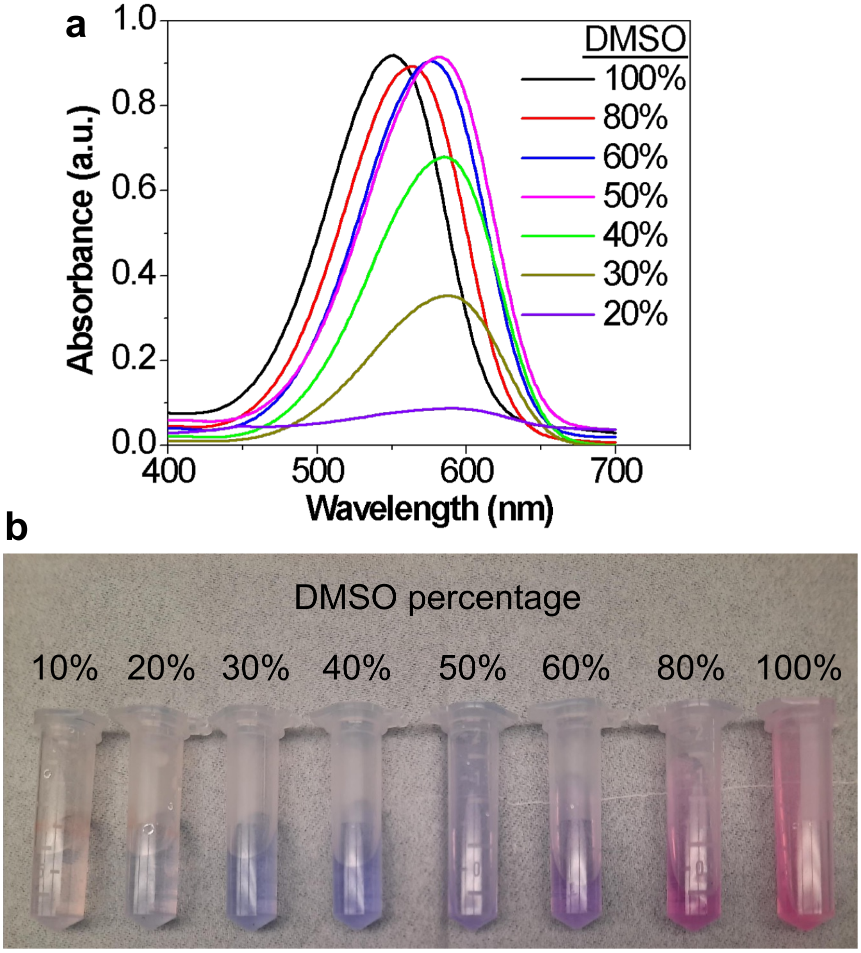

Supplementary Figure 5. Characterization of Nile red dye. a) Absorbance spectra of Nile red dye in the presence of different percentage of dimethyl sulfoxide (DMSO) in deionized water. **b)** Solubility of dye in different percentages of DMSO in deionized water.

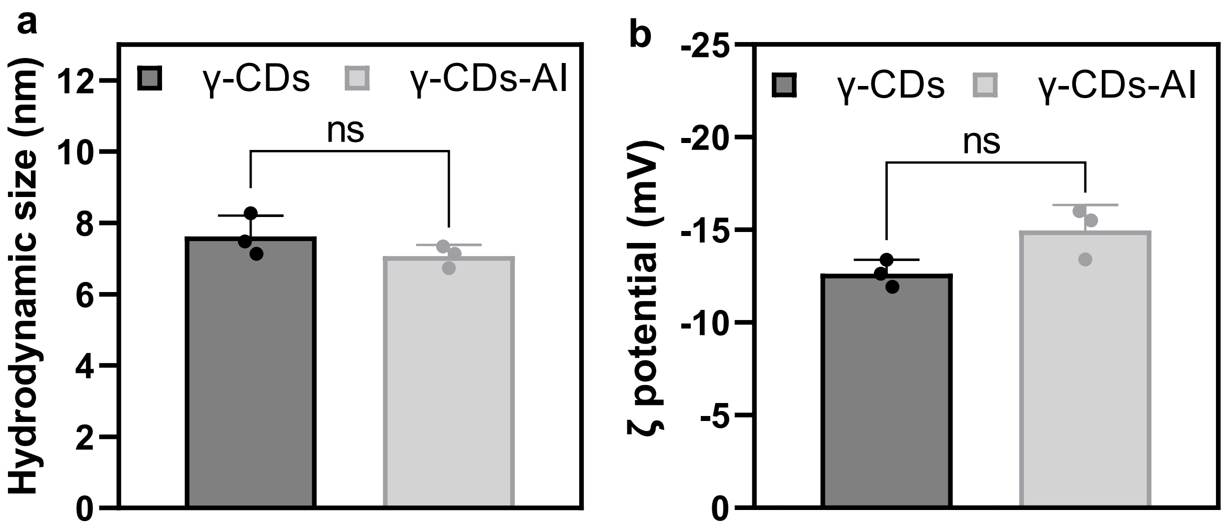

Supplementary Figure 6. Characterization of γ-CDs-AI. **a)** Hydrodynamic size and, **b)** zeta potential of γ-CDs and γ-CDs-AI (*n* = 3). γ-CDs-AI exhibited non-significant (independent t-test) change in the hydrodynamic size and zeta potential in comparison to γ-CDs, which showed efficient complexation of AI with γ-CDs.

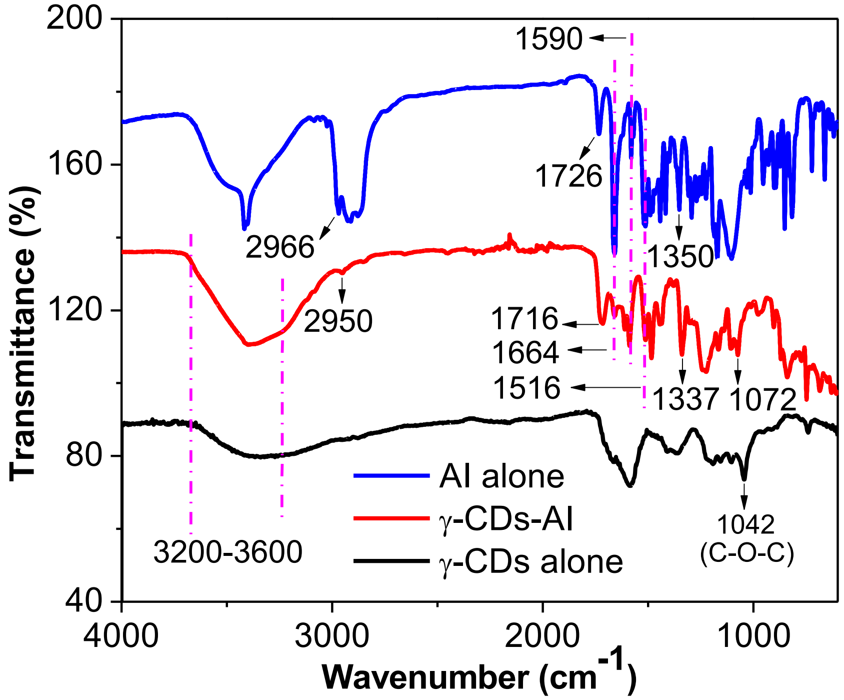

Supplementary Figure 7. Characterization of γ-CDs-AI. FTIR spectrum of γ-CDs alone, AI alone, and γ-CDs-AI. The FTIR spectrum of γ-CDs-AI showed the appearance of several new peaks that match the peaks of AI alone, in addition to the peaks corresponding to γ-CDs.

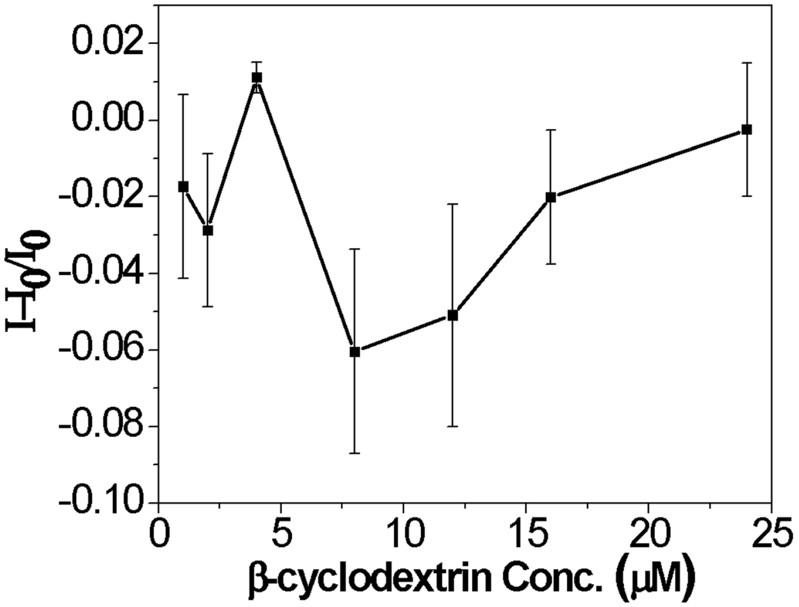

Supplementary Figure 8. No interaction between Nile red dye and β-cyclodextrins. This was indicated by no significant changes in Nile red fluorescence intensity in the presence of different concentrations of β-cyclodextrin. Values represent means and error bars indicate standard deviation (n = 3).

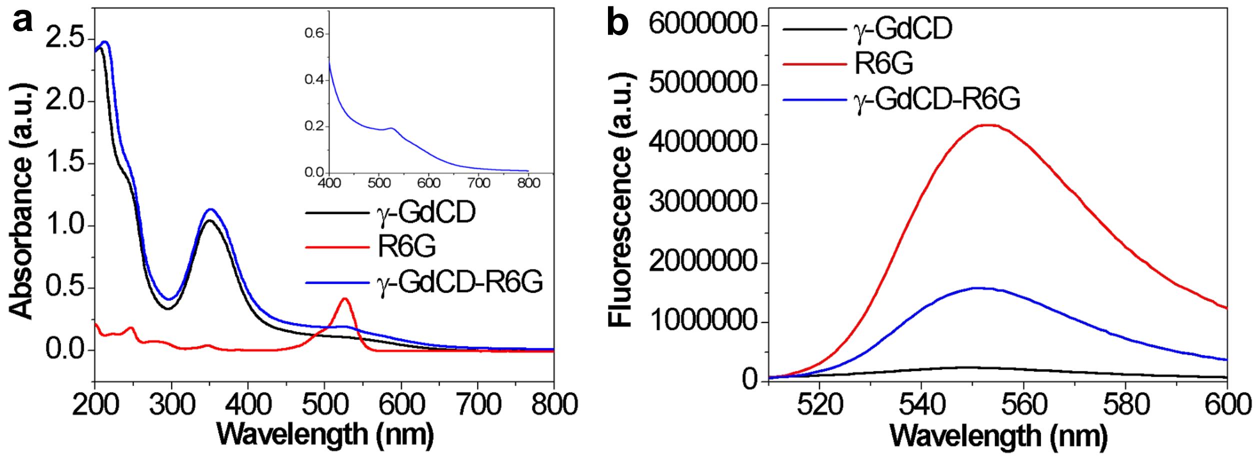

Supplementary Figure 9. Characterization of γ-GdCDs-R6G complex. a) Absorbance spectra of γ-GdCDs, R6G, and γ-GdCDs-R6G. The spectra of γ-GdCDs-R6G shows that in addition to the γ-GdCDs peak at 350 nm, a new peak at 525 nm appeared, which matches with the R6G absorbance peak (inset shows the clear R6G absorbance peak at 525 nm in γ-GdCDs-R6G). **b)** The fluorescence spectra of γ-GdCDs, R6G, and γ-GdCDs-R6G. The spectra of γ-GdCDs upon excitation at 525 nm, does not show any emission peak. In contrast, the spectra of γ-GdCDs-R6G complex showed an emission peak at 552 nm, which matches with the R6G emission peak. Together both absorbance and fluorescence measurements confirm the interaction of R6G with nanocarriers.

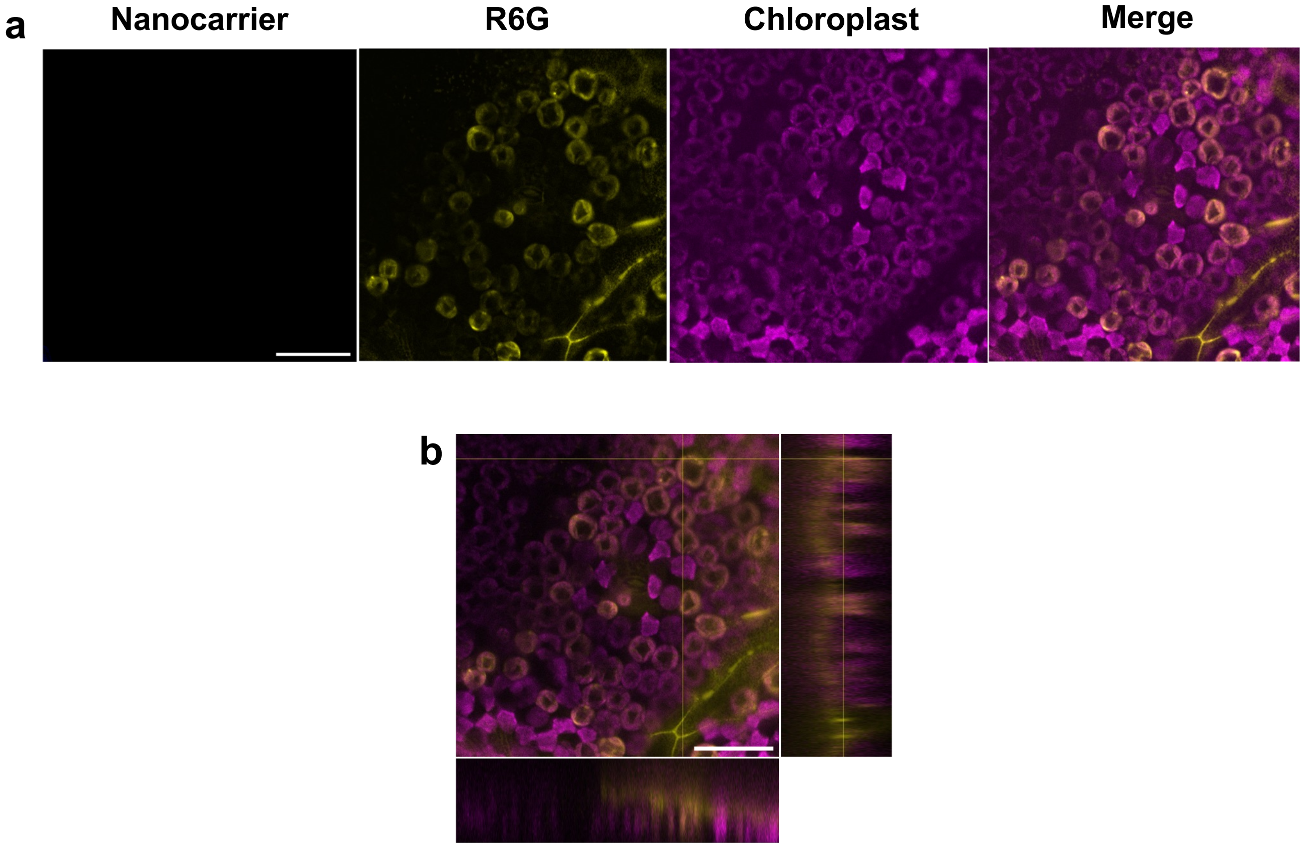

Supplementary Figure 10. Fluorescent chemical cargo (R6G) interactions with leaf surface. a) Confocal images of leaves after 3 h incubation with R6G dye. Yellow indicates R6G dye fluorescence (Ex. λ- 488 nm), magenta indicates chloroplast autofluorescence, and coral indicates the colocalization of R6G with chloroplast. **b)** Orthogonal view of confocal z-stack images of leaf treated with R6G dye. Coral color indicates the colocalization (PCC = 0.33 ± 0.02) of R6G dye with chloroplasts autofluorescence in leaf mesophyll cells, which shows that the chemical cargoes without nanocarrier can enter the soybean leaves. Scale bar = 50 μm.

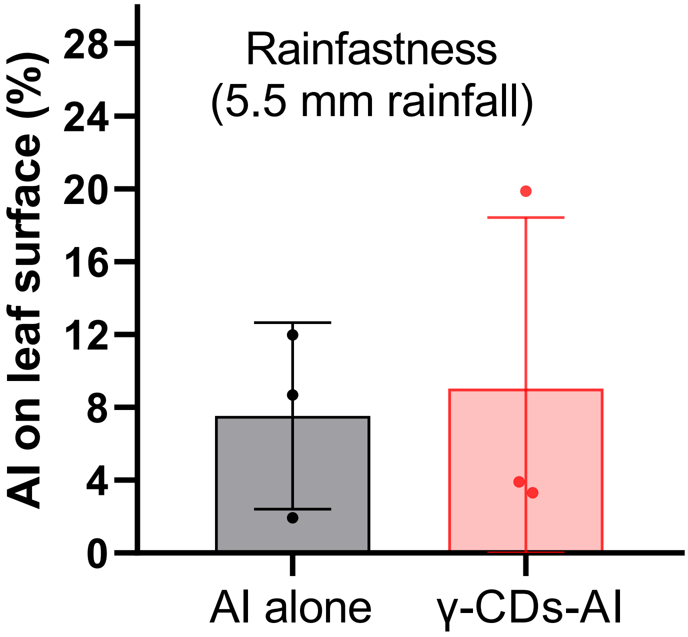

Supplementary Figure 11. Leaf surface rainfastness analysis of AI alone and Y-CDs-AI at 5.5 mm rainfall. In simulated rainfall experiment, over 90% of the AI was lost for both the nanocarrier and AI alone treatments. Values represent means and error bars indicate standard deviation (*n* = 3).

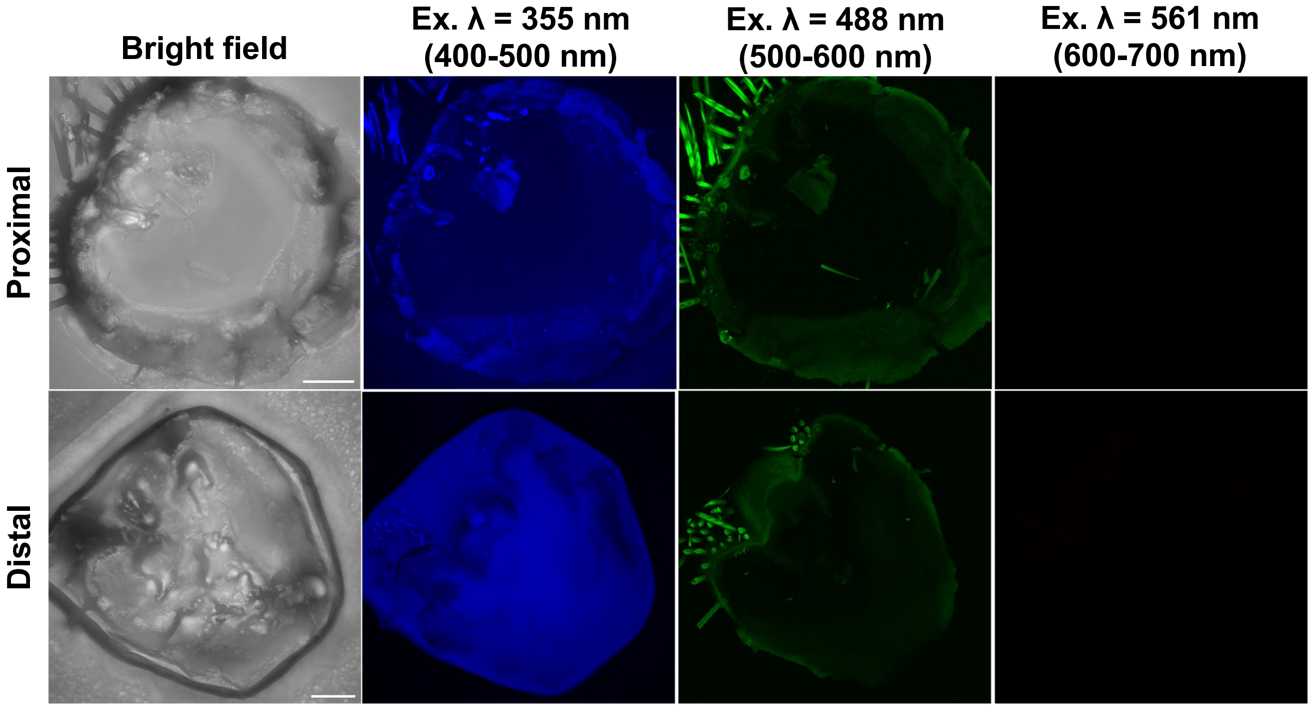

Supplementary Figure 12. Optical characterization of 50 μm tarsal sections. Confocal images of tarsal sections (T1) prepared from proximal and distal end showed autofluorescence after excitation at 355 nm and 488 nm wavelengths, but no autofluorescence after excitation at 561 nm (scale bar = 50 μm).

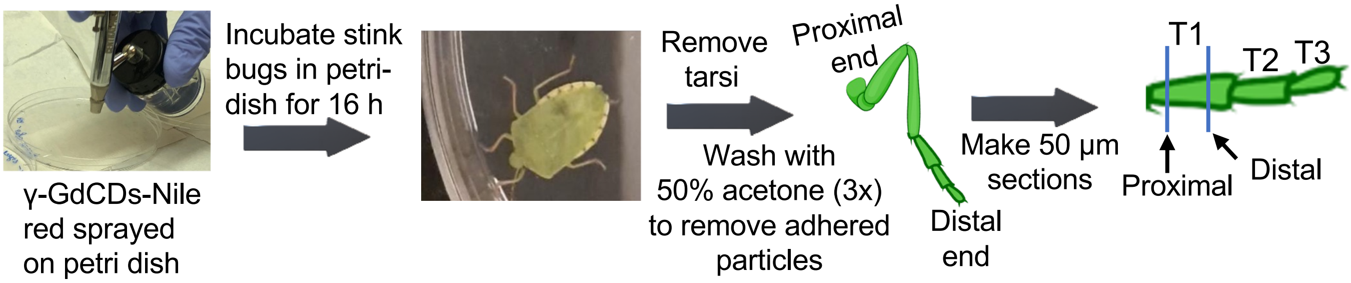

Supplementary Figure 13. Fluorescent cargo delivery to stink bugs tarsi by nanocarriers. Schematic of steps to determine the uptake of nile red delivered by γ-GdCDs nanocarriers to *N. viridula* tarsi via confocal microscopy. Created in BioRender. Lab, G. (2024) BioRender.com/x11h036

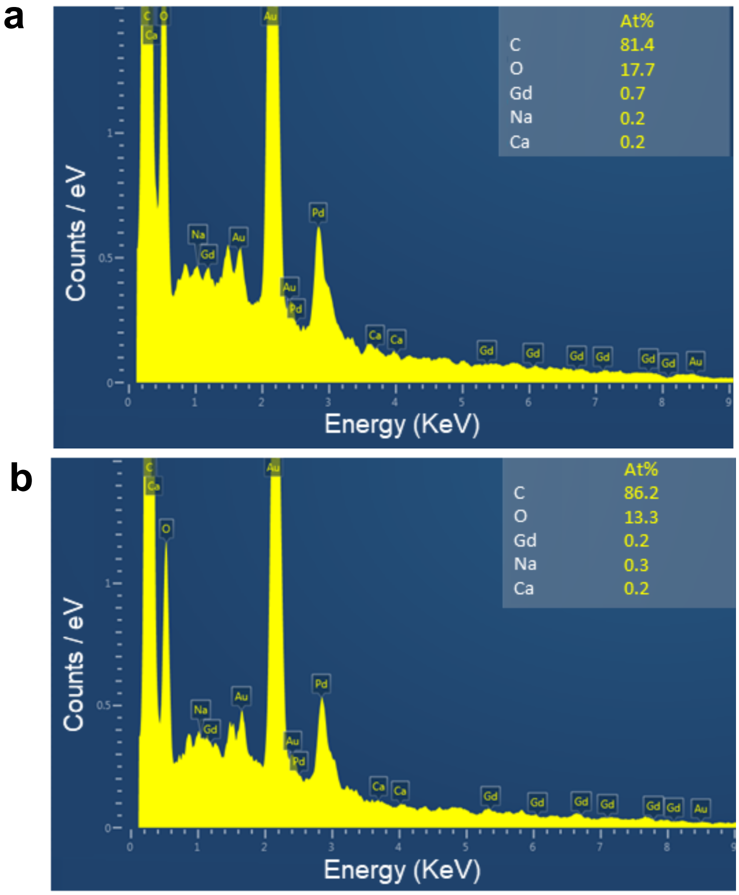

Supplementary Figure 14. Higher Gd signal from γ-GdCD nanocarriers in tarsi pore canal transects relative to no pore canals. EDX analysis performed on **(a)** pore canal transects indicated a Gd peak and a significantly higher percent of Gd atomic ratio (0.57 ± 0.12) than in (**b**) no pore canal transects (0.1 ± 0.1) ($n = 3$, $P < 0.05$).

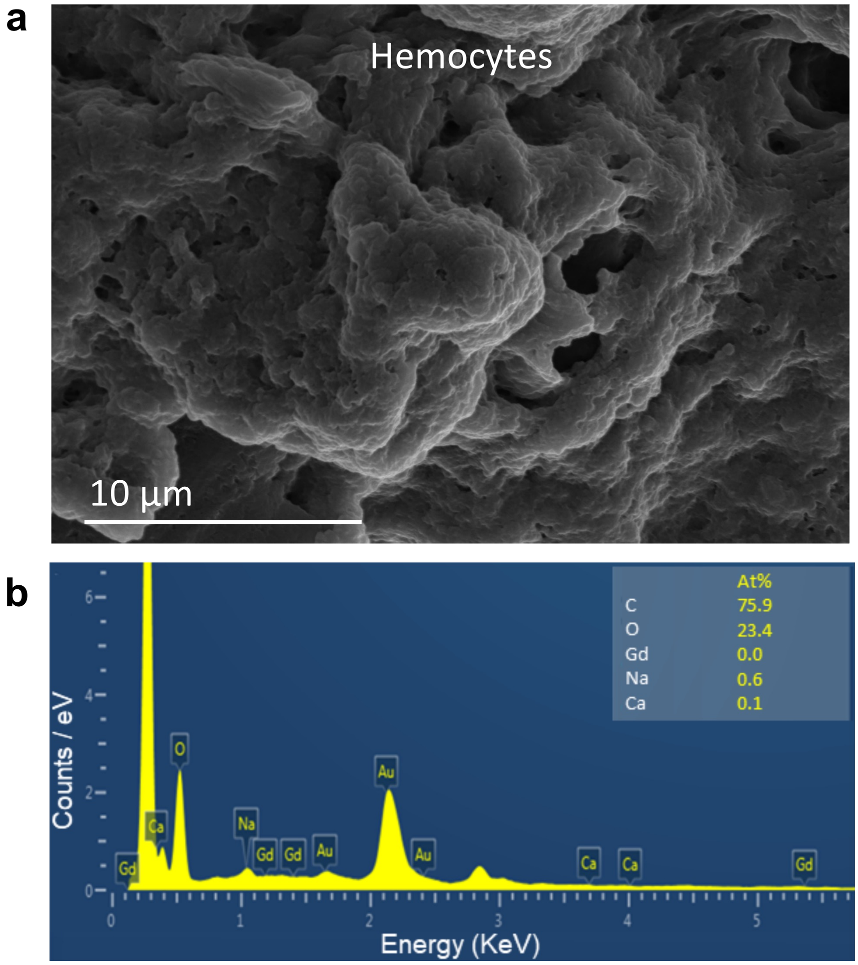

Supplementary Figure 15. No Gd detection in hemocytes from insects without γ-GdCD nanocarriers. a) SEM image of hemocytes extracted from the hemolymph collected after pricking insect's forelegs (not treated with γ-GdCDs). **b)** EDX analysis performed on the hemocytes showed no Gd signal (*n* = 3).

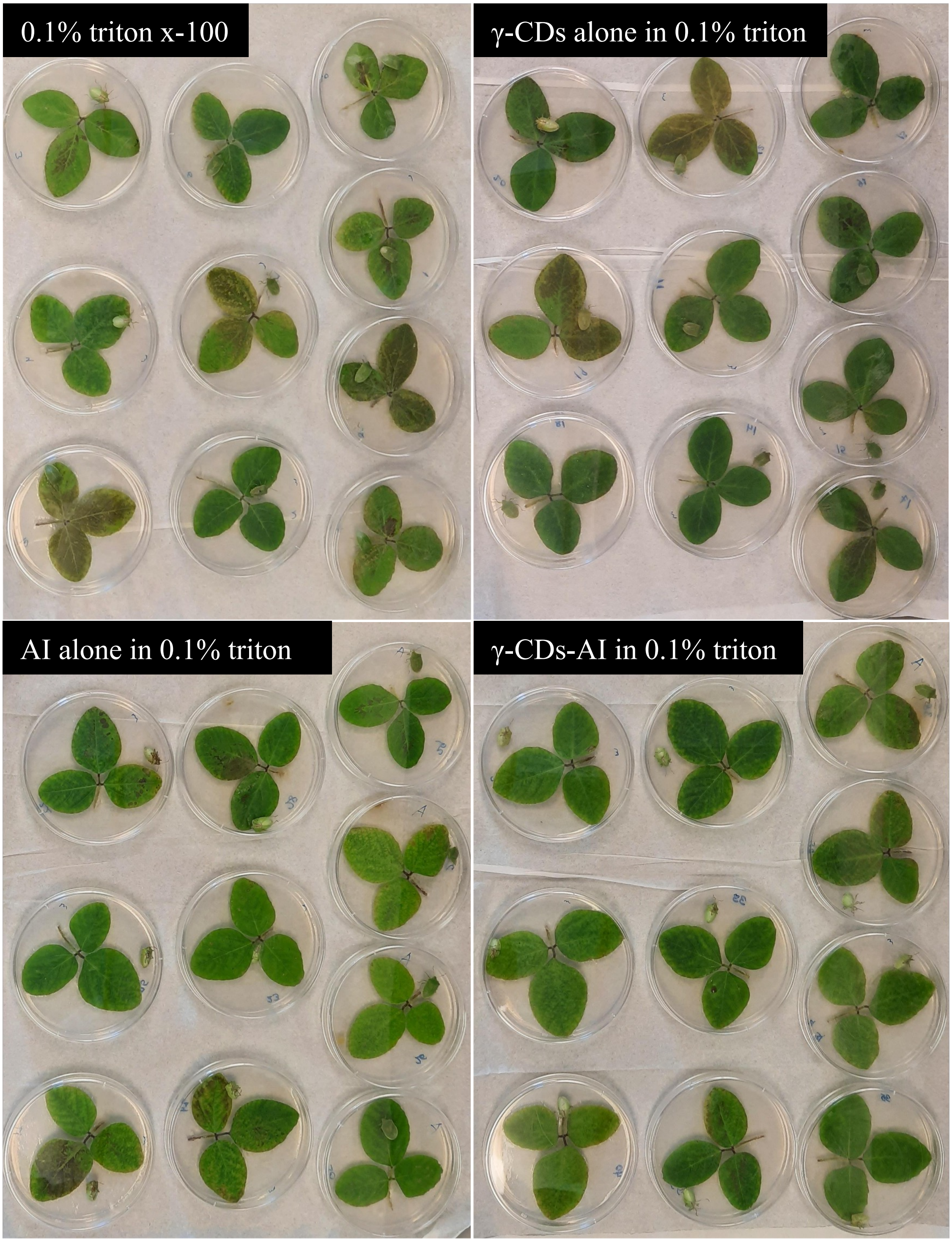

Supplementary Figure 16. Photographs of experimental set-up for insect mortality assays. Four different groups of treatment leaves were prepared by transferring the soybean trifoliate leaves of similar biometric parameters on 1% agar plates. Each group was treated with different treatments viz., 0.1% triton x-100 only, γ-CDs alone, AI alone, and γ-CDs-AI dispersed in 0.1% triton x-100. Following air-dry, the insects were randomly picked and transferred onto the treated leaves and incubated for 96 h.

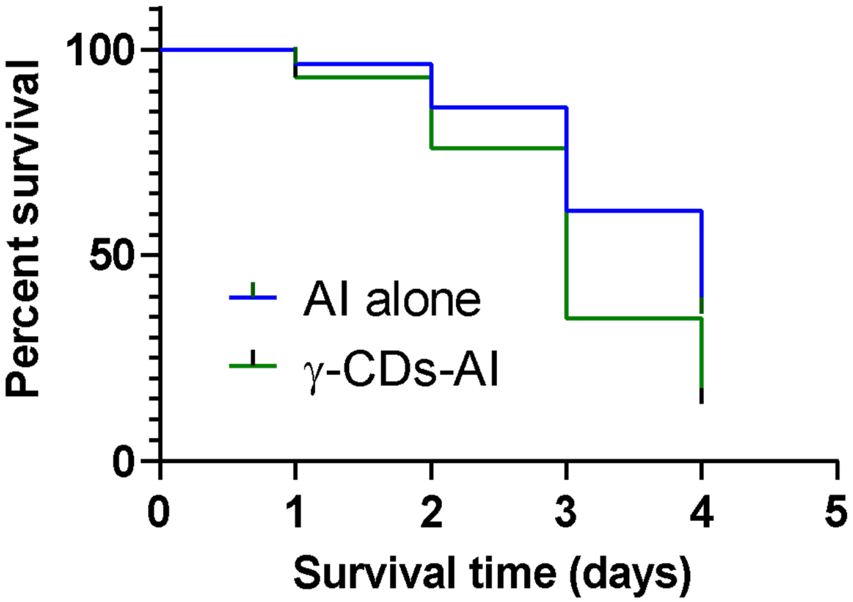

Supplementary Figure 17. Kaplan-Meier survival analysis test of stink bugs. Mantel-Cox pairwise comparison between the survival of stink bugs after treatment with AI alone and γ-CDs-AI, at 10 ppm AI concentration. The significant difference was calculated at a confidence level of 95% (*P = 0.033) using Kaplan-Meier statistics.

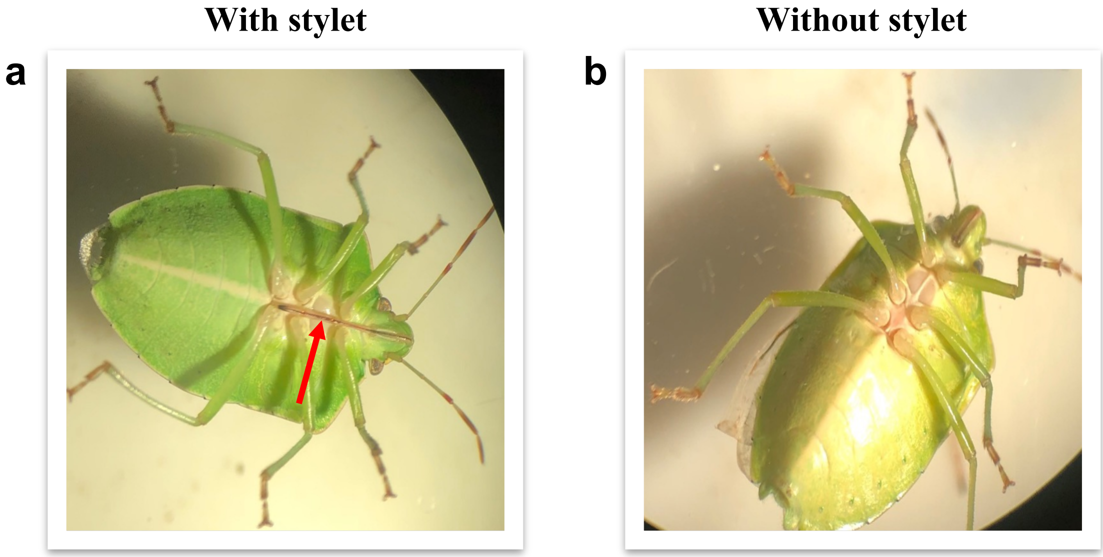

Supplementary Figure 18. Photographs of *N. viridula* ventral side. a) Photographs of stink bugs with stylet and **b)** without stylet. Red arrow in **(a)** shows the presence of needle like stylet, whereas (**b**) shows the absence of the stylet, which has been removed.

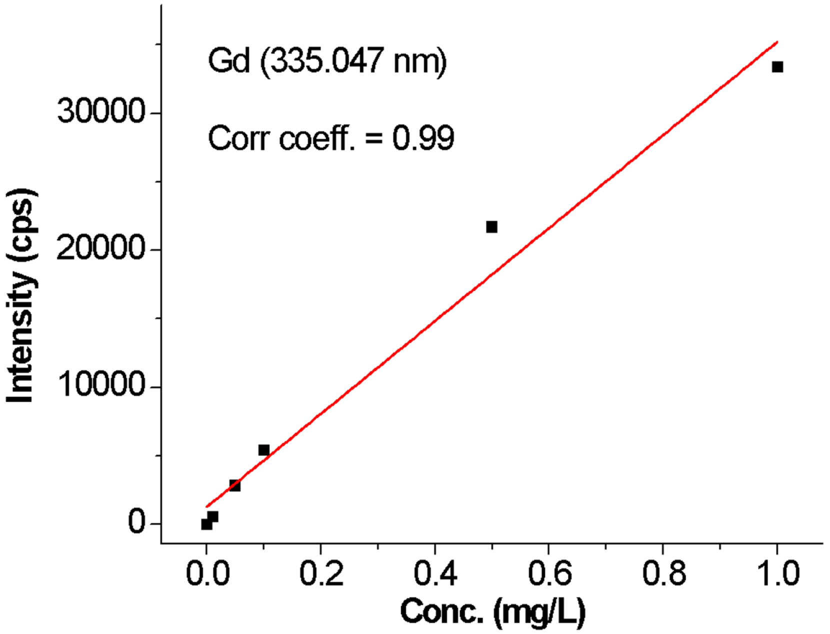

Supplementary Figure 19. Calibration curve of Gd. ICP-OES calibration curve for a range of Gd standard concentration measured at 335.047 nm wavelength. There was a linear correlation with a coefficient of 0.99.
