# Supplementary material for: Nanocarrier mediated delivery of insecticides into tarsi enhances stink bug mortality
Source: Nat Commun. 2024 Nov 11;15:9737. doi: 10.1038/s41467-024-54013-7 (PMC11554816; doi:10.1038/s41467-024-54013-7)
Supplement: Supplementary file 2 — Description of Additional Supplementary Information [file 41467_2024_54013_MOESM2_ESM.docx]

**Description of Additional Supplementary Files**

File Name: Supplementary Movie 1

Description: Tarsi proximal end treated with Nile red dye alone. The confocal fluorescence microscopy zstacks (50 µm) indicate the presence of Nile red (pink).

File Name: Supplementary Movie 2

Description: Tarsi distal end treated with Nile red dye alone. The confocal fluorescence microscopy zstacks (50 µm) indicate the presence of Nile red (pink).

File Name: Supplementary Movie 3

Description: Tarsi proximal end treated with γ-GdCDsNile red. The confocal microscopy z-stacks (50 µm) indicate the presence of Nile red fluorescent cargo (pink).

File Name: Supplementary Movie 4

Description: Tarsi distal end treated with γ-GdCDs-Nile red. The confocal microscopy z-stacks (50 µm) indicate the presence of Nile red fluorescent cargo (pink).
